# Comparison of the Effects of Prebiotics and Synbiotics Supplementation on the Immune Function of Male University Football Players

**DOI:** 10.3390/nu15051158

**Published:** 2023-02-25

**Authors:** Lufang Zhang, Hui Xiao, Li Zhao, Zeting Liu, Lanmu Chen, Chenzhe Liu

**Affiliations:** 1 Department of Exercise Physiology, School of Sport Science, Beijing Sport University, Beijing 100084, China; 2 School of China Football Sports, Beijing Sport University, Beijing 100084, China; 3 Department of Mathematic Science, School of Sport Engineering, Beijing Sport University, Beijing 100084, China

**Keywords:** prebiotic, synbiotic, upper respiratory tract infection, cytokines

## Abstract

This study was conducted to compare the effects of long-term prebiotic and synbiotic supplementations on the immunosuppression of male football players after daily high-intensity training and a one-time strenuous exercise. A total of 30 male university student-athletes were recruited and randomly assigned to the prebiotic (PG, n = 15) or synbiotic group (SG, n = 15), receiving a prebiotic or synbiotic once per day for six weeks. Physiological assessments were conducted by a maximal oxygen uptake (VO2max) test and an exhaustive constant load exercise (75% VO2max test). Inflammatory cytokine and secretory immunoglobulin A (SIgA) were measured. VO2max, maximal heart rate (HRmax), and lactic acid elimination rate (ER) were used to evaluate aerobic capacity. Upper respiratory tract infection (URTI) complaints were evaluated using a questionnaire. URTI incidence and duration were significantly lower in the SG group than that in the PG group (*p* < 0.05). At baseline, SIgA and interleukin-1β (IL-1β) levels in the SG group (*p* < 0.01) as well as IL-1β and IL-6 in the PG group (*p* < 0.05) were significantly increased, and IL-4 concentration was markedly reduced in the PG group (*p* < 0.01). The concentrations of IL-4, IL-10 and transforming growth factor-β1 (TGF-β1) were significantly reduced in the PG and SG group immediately after the constant load exercise. Significantly decreased HRmax and enhanced ER (increased by 193.78%) were detected in the SG group, not in the PG group, during the constant load experiment (*p* < 0.05) and the recovery period (*p* < 0.01), respectively. However, VO2max value was not changed. These data suggest that synbiotic supplementation for six weeks has a more positive effect than prebiotics on the immune function and athletic performance of male university football players.

## 1. Introduction

Exercise-induced immunosuppression is a common phenomenon among elite athletes after intensive exercise, which can easily increase the risk of opportunistic infection and virus reactivation [1] resulting from the changes in mucosal humoral immunity [2]. Immunosuppression in response to prolonged heavy training and match play in football is well established [3]. During this period, the risk of picking up minor infections will increase, especially an upper respiratory tract infection (URTI). Infectious pathogens enter through the mucosa of the upper respiratory tract, ultimately reducing training effects and athletic performance [4]. It has been reported that the prevalence of URTI increased in elite football players after a game [5]. Meanwhile, the level of secretory immunoglobulin A (SIgA) is decreased by 74.5% [6], which is inversely correlated with the incidence rate of URTI and thus becomes a predictor of URTI [7]. In addition, heavy training is associated with elevated numbers of T helper 2 cells (Th2) and regulatory T cells (Tregs), which produce the anti-inflammatory cytokines interleukin-4 (IL-4), IL-10, and transforming growth factor-β1 (TGF-β1). This appears to increase the risk of URTI [8]. Therefore, improving athletes’ resistance to URTI is important to guarantee high-level performance during training and competitions [9].

Probiotics are living microorganisms that can benefit the health of the host when consumed in sufficient amounts [10]. Bifidobacterium and Lactobacillus strains are the most used probiotic bacteria. Prebiotics, comprised of one or more indigestible carbohydrates, such as inulin, fructooligosaccharides (FOS), galactooligosaccharides (GOS), polydextrose (PDX), and β-glucan [11], are selectively utilized by host microorganisms and confer health benefits [12]. Synbiotics are primarily composed of probiotics and prebiotics, which can bring health benefits to the host, such as modulating gut microbiota [13], alleviating gastrointestinal symptoms [14], enhancing immunity [15], reducing inflammation and oxidative stress [16], and improving blood lipids [17]. Prebiotics and synbiotics have been shown in research to support immune defense, increase SIgA levels [18], and reduce URTI incidence and severity in healthy adults [19], high-intensity fitness professionals [20], and long-distance triathletes [21]. However, the effects of prebiotics and synbiotics on the immune function in football players during intense training and one-time strenuous exercise are poorly understood. In addition, it has been reported that prebiotics [22] and synbiotics [21] can prolong the time to exhaustion as well as promote athletic performance and aerobic capacity. In this context, the aim of the present study was to compare the effects of long-term prebiotic and synbiotic supplementation on the incidence and severity of URTI symptoms, inflammatory markers, and aerobic fitness characteristics in male university football players. The results of this study can provide an experimental basis for the application of prebiotics and synbiotics in the field of sports nutrition.

## 2. Materials and Methods

### 2.1. Participants

A total of 30 male university student-athletes from Beijing Sport University were recruited. The subjects had practiced football for 9.87 ± 0.58 years, and 86.7% of them were first-class players. The subjects were randomly assigned to the prebiotic group (PG) or synbiotic group (SG). During the experiment, the subjects maintained their normal diet. They ate at a fixed location. All subjects trained daily, with no change in schedule. The average training duration was more than 11 h/week (i.e., a high-intensity training load) [23].

### 2.2. Prebiotic and Synbiotic Administration

The prebiotic and synbiotic supplements in the present experiment were obtained from Jinhua Galaxy Biotechnology Co., Ltd. (Zhejiang, China) (the batch number for the prebiotics was 2021012101, and the batch number for the synbiotics was 2021032304). The prebiotics and synbiotics (2 g per packet) were both in powder form. The PG group was administered prebiotic supplements, namely GOS, FOS, inulin, PDX, strawberry powder, and maltitol. In addition to the same ingredients as the PG group, the SG group also contains three probiotic strains, ≥8 × 10^9^ CFU of Lactobacillus casei Zhang, Bifidobacterium lactis V9, and ≥6 × 10^9^ CFU of Lactobacillus plantarum P-8. Thus, the PG group and the SG group were regarded as the control group and experimental group, respectively. The outer packaging of the prebiotics and synbiotics was the same, and the supplements had a similar colour and smell. The subjects had to take supplements directly after lunch every day. During the experiment, the subjects were not allowed to consume any fermented food, and a two-hour time interval was maintained between taking any antibiotics and the supplements.

### 2.3. Experimental Design

**Figure 1:**
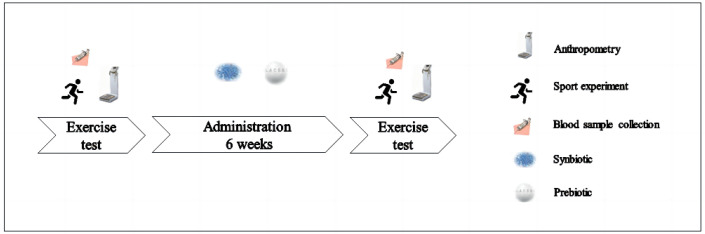
Experimental design.

The main objective of this experiment was to compare the effects of prebiotic and synbiotic supplements on the immune functions of football players after daily high-intensity training and a one-time strenuous exercise. The inclusion criteria for the subjects were as follows: no injuries during the experiment, no consumption of any prebiotics, probiotics, synbiotics, or fermented products (yogurt or other foods), and no consumption of any medications or supplements. Participants were asked to follow their regular diet for two weeks before the survey and during the programme. Prior to the experiment, all subjects were familiarised with the study process. Each participant signed an informed consent form and ensured that they could successfully complete all tests. The experimental protocol was approved by the Ethics Committee of Beijing Sport University (no. 2020167H). The participant characteristics revealed no observable differences between the SG group and PG group in terms of height, weight, age, maximal oxygen uptake (VO2max), or body mass index (BMI) (Table 1). The experimental period was six weeks. Participants had to come to the laboratory for testing before ingesting the supplement and after six weeks. The procedure and materials used were the same for the baseline and final tests. All 30 subjects completed the tests and the information collection (Figure 1).

#### 2.3.1. Anthropometry

The height of the subject was determined and recorded using a unified height metre. The fasting weight of the subject was measured using a weight tester in the morning (GMCS. RCS type IV portable).

#### 2.3.2. Upper Respiratory Tract Infection Questionnaire

During the six-week experiment, the subjects filled out the URTI questionnaire every day. The symptoms listed on the questionnaire were cold, sneezing, runny nose, cough, and sore throat [24]. The incidence and duration of URTI symptoms were considered to reflect the immune functions of the subjects during daily training.

#### 2.3.3. Mucosal Immunity

Saliva samples were gathered before and after the subjects took the supplements. The subjects were requested to sit still for 10 min before sample collection in the morning. Each subject’s head was tilted slightly forward and then to the side, and the mouth was opened for unstimulated saliva flow directly into a 5 mL plastic test tube. Samples were centrifuged and stored in a −80°C refrigerator until assaying. The levels of SIgA, β-defensin (β-DF), α-amylase (AMS), and lysozyme (LZM) were determined using commercial ELISA kits provided by Shanghai Jianglai Biotechnology Co., Ltd. (Shanghai, China).

#### 2.3.4. Maximal Oxygen Uptake

A VO2max test was conducted by adopting the classic Bruce athlete scheme for an incremental load exercise on a running platform [25]. Subjects uniformly wore an extra-small gas collection mask and heart rate (HR) band (polar V800) prior to testing. After preparing for the activity, the subjects began the test according to the experimental protocol. The subjects exercised continuously, and real-time speed, incline, and gas metabolism were accounted for using COSMED software. When the subjects reached exhaustion and could no longer carry on, the incremental load test was stopped. During the test, the experimental operator asked the subjects about their rating of perceived exertion (RPE) at each increment and monitored their HR in real time to ensure they could safely complete the incremental load test on the running platform.

To ensure that VO2max was achieved, at least three of the following criteria had to be met: (1) With an increase in exercise load, the oxygen uptake platform appears or oxygen uptake decreases; (2) with an increase in exercise load, HR does not increase; (3) respiratory quotient reaches or approaches 1.15; and (4) the RPE scale has reached the exhaustion level and can no longer maintain the current exercise load.

#### 2.3.5. Constant Load Test

To clarify the effects of prebiotic and synbiotic supplementation on inflammatory factors after prolonged aerobic exercise, the subjects warmed up for 3 min at 50% VO2max intensity and then exercised to exhaustion at 75% VO2max intensity to complete the constant load test [26]. The exercise gradient was 0. The formula used was as follows:

VO2 = 3.5 + (0.2 × speed) + (0.9 × speed × slope)

The relative values of VO2max for the PG group and SG group were 53.80 ± 1.57 mL/kg/min and 53.87 ± 1.64 mL/kg/min, respectively. During the constant load test, the warm-up speeds of the PG group and SG group were 7.12 ± 0.28 km/h and 6.85 ± 0.31 km/h, respectively, and the test speeds were 11.17 ± 0.42 km/h and 10.78 ± 0.46 km/h, respectively (Table 2). The values for HR and exercise duration were recorded, and the subjects were asked about their RPE every six minutes. The exercise protocol for the final test measurements was consistent with the protocol for the baseline tests.

#### 2.3.6. Lactic Acid Elimination Rate

Fingertip blood was collected from the subjects immediately, 1 min, 3 min, 5 min, and 10 min after the constant load test. The blood lactate level was determined using a blood lactate analyser (Biosen S_line Lab, EKF Diagnostics Holdings Ltd, Germany). Subjects remained in a seated position throughout the blood collection process and were not allowed to walk slowly, stretch, or perform other recovery activities. The lactic acid elimination rate (ER) was calculated using the following formula:

$$\mathrm{ER}=\frac{La\;(max)-La\;(10\;min)}{T\;(10\;min)-T\;(max)}$$

Here, ER is the elimination rate of blood lactic acid [27]; *La* (*max*) is the maximum value of blood lactic acid after exercise; *La* (10 *min*) is the blood lactic acid value 10 min after exercise; *T* (*max*) is the time corresponding to *La* (*max*); and *T* (10 *min*) is the 10th minute after exercise.

#### 2.3.7. Inflammatory Factors and Blood Cell Count

At baseline and immediately after the constant load exercise, 5 mL of venous blood was collected from the anterior elbow vein of each subject using standard venipuncture technology. Plasma was loaded into the green blood vessel of a sodium heparin anticoagulant for the blood cell count test. Serum loaded in the red blood collection vessel of a procoagulant was centrifuged at 4000 r/min for 10 min to remove the supernatant. The tubes were stored in a refrigerator at −80 °C until further analysis. The levels of IL-1β, IL-4, IL-6, IL-8, IL-10, TGF-β1, and IgA were determined using an ELISA kit provided by Shanghai Jianglai Biotechnology Co., Ltd. (Shanghai, China) and Wuhan Eliot Biotechnology Co., Ltd. (Hubei, China). The blood count indicators—white blood cell (WBC), red blood cell (RBC), haemoglobin level (Hb), hematocrit (HCT), mean corpuscular haemoglobin (MCH), and mean corpuscular haemoglobin concentration (MCHC)—were measured using an automatic haematology analyser (BC-2800Vet, Mindray Medical International Ltd, China).

### 2.4. Statistical Analysis

**Figure 2:**
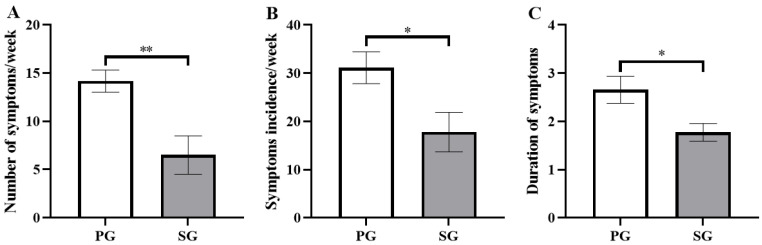
Comparison of the effects of the prebiotic and synbiotic on the upper respiratory tract infection questionnaire during high-intensity training in elite football players. Results are presented as mean ± SEM. PG: prebiotic group (n = 15); SG: synbiotic group (n = 15). (**A**) Total number of symptoms occurring in all subjects per week. (**B**) Incidence of symptomatic subjects per week. (**C**) Duration of symptoms. *: *p* < 0.05; **: *p* < 0.01.

SPSS 25 software was used to perform the analyses. The data were expressed in the form of mean ± standard error. Missing values were interpolated via expectation maximisation. Data in Table 1, Table 2, and Figure 2 were analyzed using an independent sample *t*-test. Other data were analyzed using repeated-measures ANOVA to investigate the main effects and the interactions between the group factor (prebiotic vs. synbiotic), and time factor (pre-intervention vs. post-intervention). The eta squared (η2) was also calculated to assess the effect size of the comparisons. The statistical significance level adopted was *p* < 0.05.

## 3. Results

### 3.1. Effects on URTI Symptoms

In this study, the occurrence of URTI during the six weeks of prebiotic and synbiotic supplementation was recorded (Figure 2). The total number of URTI symptoms occurring in all subjects in the SG group (6.50 ± 2.00 times/week) was significantly lower than that in the PG group (14.17 ± 1.14 times/week) (*p* < 0.01, Figure 2A). Compared to the PG group (31.11 ± 3.30%), the incidence of symptomatic subjects with URTI in the SG group was markedly lower (17.78 ± 4.10%) (*p* < 0.05, Figure 2B). In addition, the URTI duration in the SG group (1.77 ± 0.19 days/time) was significantly shorter than that in the PG group (2.66 ± 0.28 days/time) (*p* < 0.05, Figure 2C).

### 3.2. Effects on Mucosal Immunity

**Figure 3:**
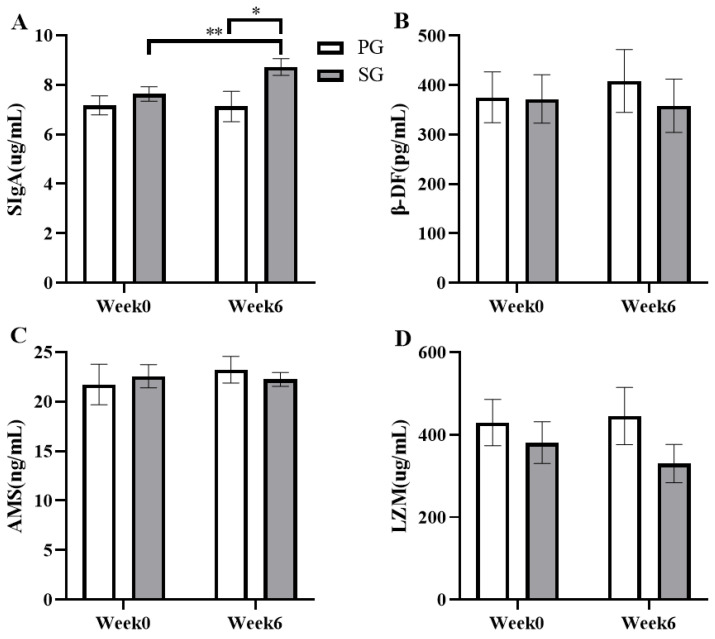
Comparison of the effects of the prebiotic and synbiotic on saliva cytokine concentration at basal status in elite football players. Results are presented as mean ± SEM. PG: prebiotic group (n = 15); SG: synbiotic group (n = 15). (**A**) SIgA concentration. (**B**) β-DF concentration. (**C**) AMS concentration. (**D**) LZM concentration. *: *p* < 0.05; **: *p* < 0.01.

The effects of six weeks of prebiotic and synbiotic supplementation on mucosal immune function during daily training were examined in this study (Figure 3). The results indicated that the level of SIgA in the SG group was significantly increased by 15.82% compared to the basal level, from 7.63 ± 0.29 pg/mL to 8.72 ± 0.34 pg/mL (*p* < 0.01, Figure 3A). The SIgA level was significantly higher in the SG group than that in the PG group in the final test (*p* < 0.05, Figure 3A). The SIgA effects showed values of *p* = 0.015 and η2 = 0.391 for the group effect, *p* = 0.001 and η2 = 0.530 for time effect, and *p* = 0.001 and η2 = 0.436 for the interaction effect. However, the PG group and SG group showed no significant differences in the levels of β-DF, AMS, and LZM in saliva (*p* > 0.05).

### 3.3. Effects on Inflammatory Markers

**Figure 4:**
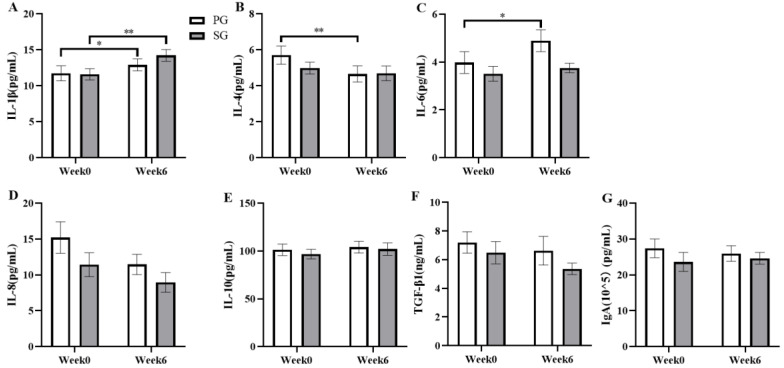
Comparison of the effects of the prebiotic and synbiotic on serum cytokine concentration at basal status in elite football players. Results are presented as mean ± SEM. PG: prebiotic group (n = 15); SG: synbiotic group (n = 15). (**A**) IL-1β concentration. (**B**) IL-4 concentration. (**C**) IL-6 concentration. (**D**) IL-8 concentration. (**E**) IL-10 concentration. (**F**) TGF-β1 concentration. (**G**) IgA concentration. *: *p* < 0.05; **: *p* < 0.01.

Changes in inflammatory factors during daily training were examined over six weeks of administering prebiotic and synbiotic supplements (Figure. 4). The results showed that the concentration of IL-1β in the SG group was increased from 11.59 ± 0.79 pg/mL to 14.22 ± 0.81 pg/mL (*p* < 0.01, Figure 4A) and increased by 13.61% (*p* < 0.05, Figure 4A) in the PG group (*p* < 0.001 and η2 = 0.455). The IL-4 level in the PG group decreased significantly from 5.70 ± 0.51 pg/mL to 4.66 ± 0.45 pg/mL (*p* < 0.01, Figure 4B) (*p* = 0.002 and η2 = 0.285). Of note, the level of IL-6 increased by 31.87% in the PG group after six weeks of supplementation (*p* < 0.05, Figure 4C) (*p* = 0.020 and η2 = 0.178). No significant changes in the serum concentrations of IL-8, IL-10, TGF-β1, and IgA were observed before or after the intervention (*p* > 0.05).

**Figure 5:**
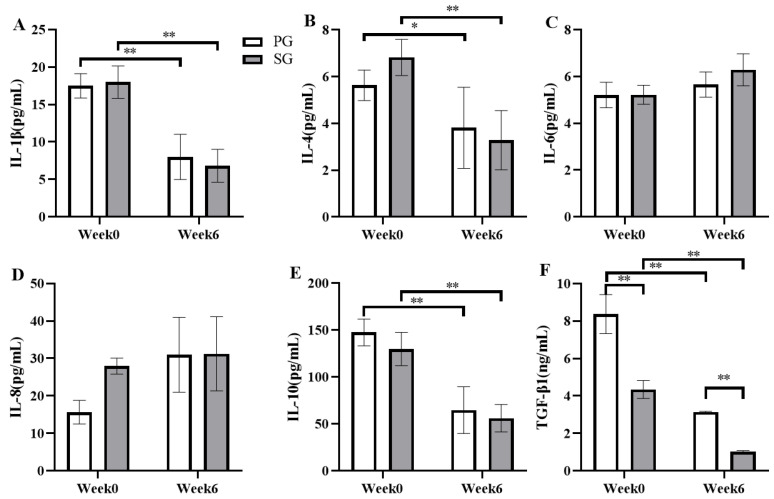
Comparison of the effects of the prebiotic and synbiotic on serum cytokine concentration immediately after the constant load test in elite football players. Results are presented as mean ± SEM. PG: prebiotic group (n = 15); SG: synbiotic group (n = 15). (**A**) IL-1β concentration. (**B**) IL-4 concentration. (**C**) IL-6 concentration. (**D**) IL-8 concentration. (**E**) IL-10 concentration. (**F**) TGF-β1 concentration. *: *p* < 0.05; **: *p* < 0.01.

Changes in inflammatory factors were also examined immediately after the constant load test (Figure 5). After six weeks of supplementation, the level of IL-1β decreased from 17.49 ± 1.63 pg/mL to 7.99 ± 3.01 pg/mL in the PG group (*p* < 0.01, Figure 5A) and reduced by 67.74% in the SG group (*p* < 0.01, Figure 5A) (*p* < 0.001 and η2 = 0.734). IL-4 decreased by 30.64% in the PG group (*p* < 0.05, Figure 5B) and reduced from 6.82 ± 0.77 pg/mL to 3.28 ± 1.27 pg/mL (*p* < 0.01, Figure 5B) in the SG group (*p* < 0.001 and η2 = 0.569). IL-10 significantly reduced by 57.93% (*p* < 0.01, Figure 5E) in the SG group and decreased from 147.56 ± 14.23 pg/mL to 64.65 ± 24.91 pg/mL (*p* < 0.01, Figure 5E) in the PG group (*p* < 0.001 and η2 = 0.734). The concentration of TGF-β1 was significantly higher in the PG group than that in the SG group in both in the baseline test (8.37 ± 1.04 ng/mL vs. 4.34 ± 0.48 ng/mL, respectively; *p* < 0.01, Figure 5F) and the final test (3.15 ± 0.03 ng/mL vs. 1.02 ± 0.07 ng/mL, respectively; *p* < 0.01, Figure 5F). The TGF-β1 concentration reduced by 53.56% in the PG group and by 74.12% in the SG group compared to the basal level (*p* < 0.01, Figure 5F). The time effect presented values of *p* < 0.001 and η2 = 0.881, the group effect showed *p* < 0.001 and η2 = 0.812, and the interaction effect of *p* = 0.015 and η2 = 0.287, whereas no significant changes were observed in the concentrations of IL-6 and IL-8 in either group (*p* > 0.05).

### 3.4. Effects on Blood Cell Counts

**Figure 6:**
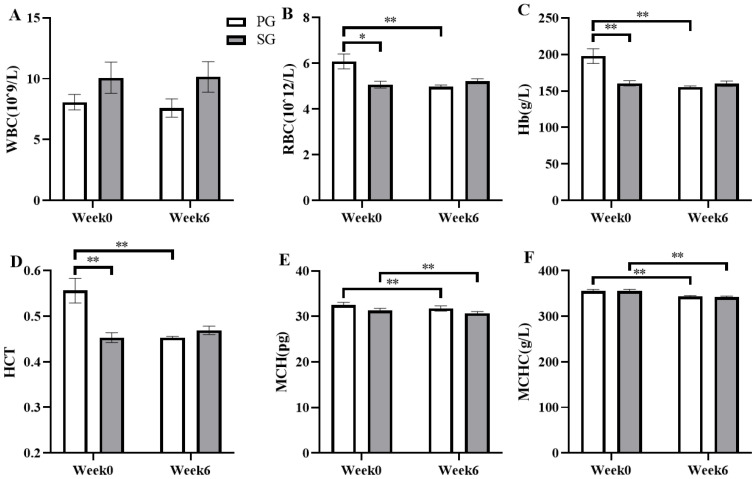
Comparison of the effects of the prebiotic and synbiotic on blood cell counts immediately after the constant load test in elite football players. Results are presented as mean ± SEM. PG: prebiotic group (n = 15); SG: synbiotic group (n = 15). (**A**) WBC concentration. (**B**) RBC concentration. (**C**) Hb concentration. (**D**) HCT concentration. (**E**) MCH concentration. (**F**) MCHC concentration. *: *p* < 0.05; **: *p* < 0.01.

After six weeks of prebiotic and synbiotic supplementation, changes in the blood cell counts were examined immediately after the constant load test (Figure 6). After the six-week intervention, the results showed that the RBC concentration was significantly decreased by 16.47% (*p* < 0.01, Figure 6B), the Hb level was markedly reduced from 197.89 ± 10.23 g/L to 155.50 ± 1.38 g/L (*p* < 0.01, Figure 6C), the HCT level was significantly decreased by 16.68% (*p* < 0.01, Figure 6D), the MCH concentration was markedly reduced from 32.52 ± 0.55 pg to 31.29 ± 0.49 pg (*p* < 0.01, Figure 6E), and the MCHC level was significantly decreased by 3.30% (*p* < 0.01, Figure 6F) in the PG group. Similarly, the levels of MCH and MCHC in the SG group were significantly decreased by 3.32% (*p* < 0.01, Figure 6E) and from 355.60 ± 3.43 g/L to 342.30 ± 2.01 g/L (*p* < 0.01, Figure 6F), respectively. The number of blood cells decreased significantly over the experiment, showing a strong effect of time (RBC: *p* = 0.009, η2 = 0.320; Hb: *p* = 0.001 and η2 = 0.460; HCT: *p* = 0.011 and η2 = 0.306; MCH: *p* < 0.001 and η2 = 0.602; MCHC: *p* < 0.001 and η2 = 0.708). In addition, a significant interaction was observed between the time and the group (RBC: *p* = 0.001, η2 = 0.446; Hb: *p* = 0.001, η2 =0.448; HCT: *p* = 0.001 and η2 = 0.453). No significant difference in WBC concentration was observed between the SG group and PG group (*p* > 0.05, Figure 6A).

### 3.5. Effects on Athletic Performance

**Figure 7:**
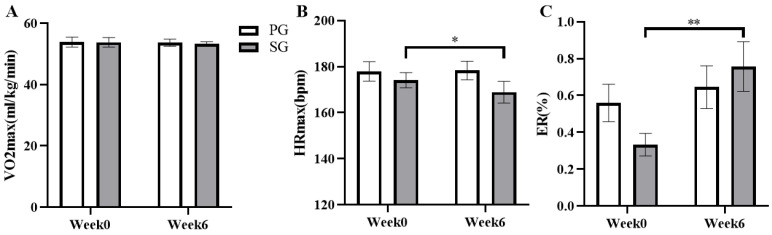
Comparison of the effects of the prebiotic and synbiotic on athletic performance during the maximal oxygen uptake and constant load test in elite football players. Results are presented as mean ± SEM. PG: prebiotic group (n = 15); SG: synbiotic group (n = 15). (**A**) VO2max. (**B**) HRmax. (**C**) ER. *: *p* < 0.05; **: *p* < 0.01.

The effects of six weeks of prebiotic and synbiotic supplementation on the athletic performance of the subjects were examined (Figure 7). The results show that there is no significant change in VO2max value in both the PG or SG group at the baseline and final test (*p* > 0.05, Figure 7A). However, the maximum heart rate (HRmax) during the constant load exercise was significantly reduced from 174.10 ± 3.35 bpm to 168.88 ± 4.70 bpm in the SG group (*p* < 0.05, Figure 7B) (*p* = 0.011 and η2 = 0.364). In addition, ER was significantly increased by 193.78% compared to the basal level (from 0.33 ± 0.06% to 0.76 ± 0.13%) during the recovery period after exercise in the SG group (*P* < 0.01, Figure 7C) (*p* = 0.013 and η2 = 0.295).

## 4. Discussion

The results of this study revealed that synbiotic supplementation reduced the incidence and duration of URTI, increased the levels of SIgA and IL-1β in male university football players during high-intensity training, and decreased the concentrations of IL-4, IL-10, and TGF-β1 immediately after the constant load test. The prebiotic supplementation reduced IL-4 and increased IL-1β and IL-6 at baseline as well as decreased IL-4, IL-10, and TGF-β1 levels after prolonged aerobic exercise. Additionally, increased ER in prolonged aerobic exercise were observed in the synbiotic group, not in the prebiotic group.

It is reported that prebiotics and synbiotics contribute to reducing the incidence and severity of URTI [28,29]. Dharsono et al. [19] and Auinger et al. [28] reported that taking prebiotic β-glucan enhances the immune functions of healthy adults and reduces the incidence, duration, and severity of URTI. In addition, McFarlin et al. [30] found that marathon runners have a significantly lower incidence of URTI after taking prebiotic β-glucan. It is also important to note that Bergendiova et al. [31] observed a significant reduction in the incidence of URTI symptoms when athletes who engaged in kayaking, mountain biking, swimming, shooting, running, and cycling were supplemented with prebiotic β-glucan. Hor et al. [32] proved that Lactobacillus casei Zhang, a probiotic component of the synbiotic used in this experiment, can alleviate URTI symptoms in healthy adults, reduce the duration of nasal, pharyngeal, and general flu as well as total respiratory illness symptoms, thus preventing sickness from strenuous exercise and increasing the chances of staying healthy. A previous study revealed a significant reduction in the number and duration of URTI episodes in healthy adults after supplementation with synbiotics [29]. In the present study, the incidence and duration of URTI were lower in the SG group than that in the PG group during six weeks of supplementation. It is suggested that supplementation with synbiotics rather than prebiotics has the ability to reduce the incidence and duration of URTI in football players.

Salivary SIgA, the main type of antibody found in mucosal secretions, [33] along with β-DF [34], AMS [35], and LZM [36] can inhibit pathogen colonization. Reduced secretion of SIgA is a risk factor for the development of URTI in physically active individuals [23]. Low resting salivary IgA concentration has been reported in some elite athletes [37]. Prebiotics and synbiotics have been reported to act directly on mucosal immune cells to promote the secretion of SIgA, thereby inhibiting the growth of pathogens and regulating immune function. Xu et al. [38] reported that administering the same probiotic used in this experiment could increase serum IgG and faecal SIgA levels in dogs. The present study revealed a significant increase in the SIgA level of football players after six weeks of synbiotic supplementation and markedly higher in the SG group than that in the PG group in the final test. These results are consistent with Coman et al.’s [20] findings that synbiotic supplementation can significantly increase the salivary SIgA levels of high-intensity fitness professionals. In addition, Childs et al. [39] observed that the level of salivary IgA in healthy adults increased significantly after synbiotic supplementation. In contrast, prebiotic supplementation had no significant effect on SIgA in this study. Therefore, the results of this study suggest that synbiotics may enhance respiratory mucosal immune function more than prebiotics by increasing SIgA level.

T cells belong to the adaptive immune system and are essential for coordinating the immune response to invading and existing pathogens. T cells can be divided into three subgroups based on their polarised phenotypic characteristics: Th1, Th2, and Tregs [8]. Th1 cells drive cell-mediated immunity and play an important role in the defense against viral infections [40]. A reduction in the proinflammatory cytokines IL-1β, IL-6, and IL-8 produced by Th1 cells may increase the risk of infection and virus reactivation. It has been reported that high levels of Th2 cytokines are found in the bodies of athletes prone to disease [23]. Anti-inflammatory concentrations of IL-4, IL-10, and TGF-β produced by Th2 and Tregs can inhibit Th1 cell function and immune response [41]. Childs et al. [39] reported that synbiotics can promote Th1 response and reduce Th2 activity, thus improving the immune function of the body. Similarly, Childs et al. [39] observed that administering the prebiotic xylooligosaccharide to healthy adults resulted in a significant decrease in the level of the anti-inflammatory cytokine IL-10. The previous report [42] found the synbiotic increased concentration of IL-1β. In this experiment, the IL-1β level was significantly increased at baseline and the levels of IL-4, IL-10, and TGF-β1 were decreased upon prolonged aerobic exercise in the SG group. Decreased levels of IL-4 in the base state as well as IL-4, IL-10, and TGF-β1 after aerobic exercise were detected in the PG group. Therefore, synbiotics and prebiotics may not make a significant difference in reducing anti-inflammatory cytokines and increasing pro-inflammatory cytokines to improve immune function in athletes after daily training and a single bout of exercise.

WBC forms an important part of the immune system [43]. However, the results of the present study showed that six weeks of prebiotic and synbiotic supplementation had no significant effect on the WBC count of athletes. This is in agreement with Childs et al.’s report [39] that no significant changes in leukocytes were observed after administering prebiotics and synbiotics to healthy adults. Therefore, the results of this study suggest that both prebiotic and synbiotic supplementation may not have a significant difference in the effect of WBC in football players after a single strenuous exercise.

VO2max reflects the body’s ability to inhale, transport, and utilise oxygen and is thus one of the most significant indicators of the human body’s aerobic capacity [44]. Blood indicators to assess oxygen transport capacity include RBC, Hb, HCT, MCH, and MCHC, which have similar patterns of variation [45]. In this study, both the PG group and SG group showed significant decreases in MCH and MCHC after the constant load experiment, but the decrease was more pronounced in the PG group. It is suggested that synbiotics can counteract the adverse effects of a one-time strenuous exercise and have positive results on blood health. This aligns with Farinha et al.’s [46] results, which showed that significant increases in MCH and MCHC promoted positive changes in blood health. Although supplementation with prebiotics and synbiotics did not affect VO2max level in the subjects, a significant increase in ER was observed in the SG group compared to the PG group, suggesting that the ability to metabolize lactic acid is increased in the SG group. Furthermore, consistent with the previous result that the HRmax is significantly decreased in road cyclists after probiotic supplementation [47], obviously decreased HRmax was found in the SG group during the constant load experiment. Therefore, supplementation with synbiotics may help athletes maintain their exercise performance by promoting lactate metabolism and enhancing aerobic capacity. Salarkia et al. [48] and Lin et al. [49] reported that the improved aerobic capacities and athletic performance of female swimmers and middle-distance runners may be due to the increased resistance to URTI and the improved immune function resulting from supplementation [50]. As a consequence, the reduced incidence of URTI and enhanced immune function observed in the SG group may also be due to the factors that enhance athletic performance. However, the limitation of our study is that there was no placebo control group. In the future study, this should be taken into account.

## 5. Conclusions

In conclusion, supplementation with synbiotics is better than prebiotics at improving immune function in football players by reducing the incidence and duration of URTI and increasing SIgA level. In addition, synbiotics have a more beneficial effect than prebiotics on improving lactate metabolism and exercise performance.

## Figures and Tables

**Table 1:** Study participant characteristics.

Variable	PG (n = 15)	SG (n = 15)	* *p*-Value
Height (cm)	178.98 ± 1.98	178.18 ± 1.86	0.782
Weight (kg)	70.13 ± 2.14	72.75 ± 1.95	0.375
BMI (m^2^/kg)	22.13 ± 0.63	23.07 ± 0.56	0.280
VO2max (mL/kg/min)	53.87 ± 1.64	53.80 ± 1.57	0.977
Age (years)	20.40 ± 0.31	20.20 ± 0.20	0.821
Years training (years)	10.27 ± 0.84	9.47 ± 0.82	0.501

Results are expressed as the mean (SEM) before the experiment. All reported *p* values are based on comparisons between PG and SG. BMI: body mass index; VO2max: maximal oxygen uptake. *: *p* < 0.05.

**Table 2:** Summary information of the constant load experiment.

Variable	PG (n = 15)	SG (n = 15)	* *p*-Value
Warm-up speed (km/h)	7.12 ± 0.28	6.85 ± 0.31	0.521
Movement speed (km/h)	11.17 ± 0.42	10.78 ± 0.46	0.538
Exhausted exercise time (min)	37.30 ± 4.32	53.20 ± 6.97	0.068

Results are represented as the mean (SEM) before and after the experiment. All reported *p* values are based on comparisons between PG and SG. *: *p* < 0.05.

## Data Availability

The data presented in this study are available on request from the corresponding author. The data are not publicly available due to the subject’s privacy and confidentiality.
